# Federated Learning: Breaking Down Barriers in Global Genomic Research

**DOI:** 10.3390/genes15121650

**Published:** 2024-12-22

**Authors:** Giulia Calvino, Cristina Peconi, Claudia Strafella, Giulia Trastulli, Domenica Megalizzi, Sarah Andreucci, Raffaella Cascella, Carlo Caltagirone, Stefania Zampatti, Emiliano Giardina

**Affiliations:** 1 Genomic Medicine Laboratory UILDM, IRCCS Santa Lucia Foundation, 00179 Rome, Italy; 2 Department of Science, Roma Tre University, 00146 Rome, Italy; 3 Department of Systems Medicine, Tor Vergata University, 00133 Rome, Italy; 4 Department of Biomedicine and Prevention, Tor Vergata University, 00133 Rome, Italy; 5 Department of Chemical-Toxicological and Pharmacological Evaluation of Drugs, Catholic University Our Lady of Good Counsel, 1010 Tirana, Albania; 6 Department of Clinical and Behavioral Neurology, IRCCS Santa Lucia Foundation, 00179 Rome, Italy

**Keywords:** federated learning, artificial intelligence, machine learning, precision medicine, genomic data privacy, NGS sequencing

## Abstract

Recent advancements in Next-Generation Sequencing (NGS) technologies have revolutionized genomic research, presenting unprecedented opportunities for personalized medicine and population genetics. However, issues such as data silos, privacy concerns, and regulatory challenges hinder large-scale data integration and collaboration. Federated Learning (FL) has emerged as a transformative solution, enabling decentralized data analysis while preserving privacy and complying with regulations such as the General Data Protection Regulation (GDPR). This review explores the potential use of FL in genomics, detailing its methodology, including local model training, secure aggregation, and iterative improvement. Key challenges, such as heterogeneous data integration and cybersecurity risks, are examined alongside regulations like GDPR. In conclusion, successful implementations of FL in global and national initiatives demonstrate its scalability and role in supporting collaborative research. Finally, we discuss future directions, including AI integration and the necessity of education and training, to fully harness the potential of FL in advancing precision medicine and global health initiatives.

## 1. Introduction

In recent years, advancements in NGS technologies have revolutionized genomics, offering new insights into the structure, function, and variability of genetic material. NGS enables the simultaneous sequencing of millions of DNA fragments, crucial for applications such as genome assembly, transcriptomics, epigenomics, and metagenomics [1]. Various NGS platforms such as Illumina, Pacific Biosciences (PacBio), and Oxford Nanopore operate on sequencing principles that influence generated data types and their applications. Short-read platforms like Illumina are widely used for high-throughput sequencing due to their cost-effectiveness and accuracy, making them suitable for detecting small-scale variations such as single-nucleotide polymorphisms (SNPs). On the other hand, long-read technologies like PacBio and Nanopore can detect complex genomic structures, such as large insertions, deletions, and structural rearrangements, which are essential for understanding epigenetic modifications and resolving repetitive sequences [2].

This variety of platforms leads to heterogeneity in genomic data among institutions, as datasets differ in their sequencing depth, read lengths, error profiles, and bioinformatics processing pipelines [3]. This variability results from each laboratory’s choice of technology and analytical approaches, which are fitted to their specific goals, but complicate the integration and standardization of genomic data. Each research or clinical center may prioritize specific NGS technologies, such as whole-genome sequencing (WGS), whole-exome sequencing (WES), or targeted sequencing panels. While the growth of genomic data holds could encourage discoveries in precision medicine and population genetics, it raises critical issues regarding data privacy, standardization, and equitable access. Without data-sharing frameworks, discrepancies in sequencing platforms, data processing, and population-specific datasets create barriers to collaboration, limiting the scalability and generalizability of genomic research [1]. Another significant issue is the existence of data silos, where genomic and clinical data are isolated throughout specific institutions, making it difficult to aggregate, analyze, and share the data [4,5]. Data silos arise from a combination of technical, organizational, and regulatory factors. The heterogeneity of platforms and practices contributes significantly by complicating integration efforts, while additional barriers such as data privacy regulations and institutional policies exacerbate the challenge. Genetic data, due to its potential to uniquely identify individuals and reveal information about their families, raises serious privacy concerns regarding data protection, especially in light of stringent regulations such as the GDPR, which governs how personal data can be processed and shared across jurisdictions [6].

In this context, FL is a promising solution to data fragmentation and privacy concerns. FL is an innovative machine-learning approach that allows institutions to collaborate on data analysis while keeping the data decentralized. Instead of transferring raw data across centers, institutions share only model updates, which are aggregated to improve the global model. This process ensures that sensitive data from hospitals, research institutions, or other organizations remain at their source, without compromising privacy regulations like GDPR. FL offers several key advantages, especially in critical data privacy fields [7]. FL reduces the risk associated with data violation and ensures that data control is respected, allowing institutions to train machine learning models without sharing the raw data. This decentralized approach resolves the issue of silos by enabling the extraction of insights from distributed datasets without physically aggregating the data. Thus, it promotes cross-institutional research, facilitates collaboration, and accelerates the development of precision medicine and genomic-based innovations. Furthermore, integrating FL into national and international genomic networks, such as the Global Alliance for Genomics and Health (GA4GH) or the European Genome-phenome Archive (EGA), can amplify its potential by promoting greater data sharing and harmonization. By utilizing both technical and regulatory challenges, these frameworks help integrate different datasets, allowing researchers to overcome barriers related to institutions and locations. As genomic data continue to grow in complexity and volume, FL will play an essential role in surpassing the barriers of data privacy and fragmentation, offering a way to break down silos and unlock the full potential of global genomic collaboration [8].

Although FL has been extensively studied in healthcare, its application in genomics presents unique challenges and opportunities that remain underexplored. Recent reviews have addressed FL in healthcare broadly, but significant issues remain. For example, Crowson et al. conducted a systematic analysis of FL applications in oncology and radiology, identifying significant gaps in data quality and model bias. However, their work primarily focuses on imaging and clinical prediction tasks, avoiding analyses of the complexities of genomic data [9]. Similarly, Cremonesi et al. discuss multimodal data modelling for FL healthcare platforms, highlighting interoperability challenges. While comprehensive, their review centers on data integration frameworks rather than the specific technical and regulatory barriers in genomics [10]. Lastly, Chowdhury et al. emphasize FL applications in cancer research but lack detailed insights into how FL can address challenges such as heterogeneous sequencing technologies or the strict privacy requirements of genomic data [11]. This review addresses these gaps by focusing on FL applications in genomics. Specifically, it tackles the unique heterogeneity of sequencing platforms and the need for standardization across genomic datasets, providing actionable insights into overcoming these technical barriers. In addition, it offers an in-depth analysis of FL’s ability to be compliant with stringent privacy regulations, such as GDPR, through advanced mechanisms like differential privacy and secure aggregation protocols, enabling collaborative genomic research without compromising data confidentiality. The review further highlights FL’s role in amplifying the impact of international genomic initiatives, exploring how FL can foster global collaboration and data harmonization. By addressing these genomics-specific challenges and providing actionable insights, this review aims to pave the way for more effective and collaborative genomic research, ultimately advancing precision medicine.

## 2. The Promise of Federated Learning in Genomics

FL is a decentralized machine learning approach that allows multiple institutions or devices to train a model collaboratively, without the need to transfer sensitive data to a central server [12]. In the context of genomics research, this method enables the analysis of data from the sources, keeping it within the confines of each organization or specific region. This is particularly crucial in fields where privacy and data protection are essential, such as genomic research involving personal health data [13]. The transition from centralized data models to federated architectures represents a critical change in how genomic data are shared and analyzed. Conventionally, data centralization involves the transfer of information, which raises concerns about privacy and compliance issues with regulations. However, FL brings the code to the data rather than moving it, ensuring that institutions can still benefit from shared insights without compromising rigorous privacy standards [14] (Table 1).

While FL provides a robust framework for decentralized data analysis, existing infrastructures like ELIXIR contribute significantly to secure and distributed genomic research, even though they do not rely on FL [15]. ELIXIR is one of the leading infrastructures for genomics in Europe. It facilitates access to genomic and clinical data from institutions across the continent, ensuring that data remain within local jurisdictions. ELIXIR promotes secure data sharing and collaboration among researchers while complying with local privacy laws. Its centralized architecture allows data to be stored and accessed in compliance with regulations such as the GDPR, but without physically transferring data across borders. This approach demonstrates how distributed data management can help research collaboration and ensure privacy, compliant with regulations, despite its difference from federated learning.

FL is changing how we manage genomic information, driving advancements in personalized healthcare while maintaining stringent privacy protections. FL simplifies the process by enabling data analysis without data transfer, which helps reduce administrative complexities and logistical obstacles while improving computational efficiency. This approach allows researchers to collect data insights from various centers, enhancing research efforts’ overall scalability and speed [16].

## 3. Federated Learning Methodology

FL can be categorized into three primary types: Horizontal Federated Learning (HFL), Vertical Federated Learning (VFL), and Federated Transfer Learning (FTL). Each is suited to different data distribution scenarios [17]. These approaches play distinct roles in enabling secure and collaborative genomic research while addressing privacy concerns and regulatory constraints. HFL is most applicable when datasets across institutions share the same features but include different samples. This scenario is common in genomic studies where institutions collect similar genetic markers or sequencing data from diverse populations. HFL allows for the decentralized training of machine learning models, leveraging data from multiple sites without sharing sensitive genomic information. This makes HFL particularly suited for population-scale studies, such as genome-wide association studies (GWAS), where data silos otherwise hinder collaboration [18]. VFL comes into play when datasets share overlapping samples but have distinct features. This approach is valuable in scenarios where genomic data from one institution can be combined with phenotypic or clinical data from another. VFL enables the integration of such complementary datasets, providing richer insights while maintaining data privacy [19]. For example, linking genotypic data to clinical outcomes can enhance precision medicine initiatives. FTL is used when datasets differ in both samples and features. FTL is beneficial for transferring knowledge between regions or populations with distinct genomic characteristics. This can be critical in adapting models trained on European genomic datasets for application to Asian or African populations, facilitating global equity in genomic research [20]. Among these, HFL is most widely applicable to genomics due to the prevalence of homogenous feature sets in distributed datasets. However, VFL is invaluable for multi-omics studies and clinical-genomic data integration, while FTL holds promise for addressing underrepresentation in genomic research. By selecting the appropriate type of FL, genomic research can harness the benefits of collaborative data analysis, overcoming privacy and regulatory challenges while advancing precision medicine and global health initiatives.

**Figure 1:**
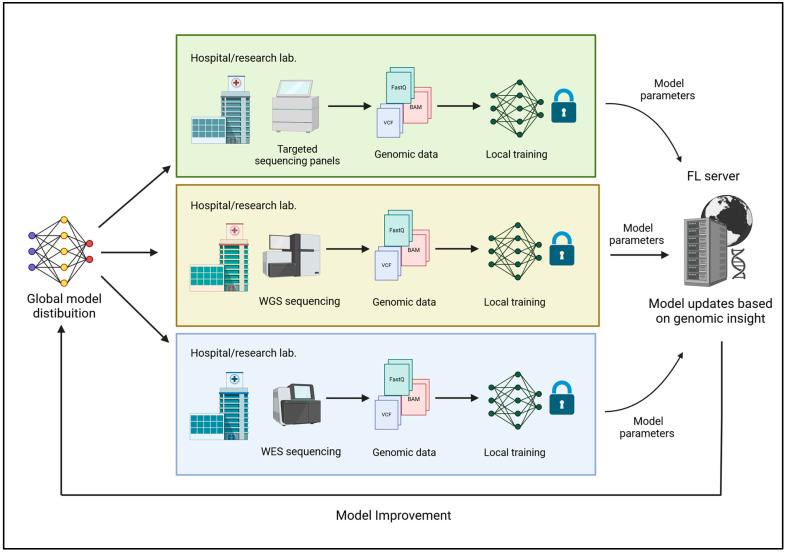
Federated Learning process in genomic research: institution-specific genomic data (e.g., FASTQ, BAM, VCF) from diverse sequencing platforms is used to train local models. Model updates are securely aggregated at a central server to create an improved global model, which is redistributed to institutions for further analysis, ensuring privacy, scalability, and continuous model enhancement.

Regardless of the specific type of FL applied, the methodology ensures that sensitive genomic data remain protected. During the initial stage of Local Data Storage and Model Training, each institution (such as a hospital or research laboratory) stores its data locally, training machine learning models on site rather than transferring raw data to a central server. This decentralized approach safeguards sensitive information and prevents physical data transfers that could expose the data to breaches. However, challenges persist in securely managing local data storage and ensuring the reliability of locally trained models. A critical consideration in genomic research is how the data are partitioned. Genomic data can be partitioned by a population or sequencing platform, impacting the model’s performance [21,22]. For instance, partitioning by population enables the creation of training models specific to a given demographic, but it may introduce challenges in aggregating knowledge from diverse groups. Partitioning by sequencing platforms, such as using data from different technologies like Illumina and PacBio, can further complicate model aggregation due to the inherent variability in the data across platforms. These challenges emphasize the need for effective model aggregation strategies to preserve collective knowledge while maintaining privacy. Once the local models are trained, the second stage involves Aggregation of Model Updates. The model updates, consisting of the model parameters adjusted based on the local data, are sent to a central server for aggregation. This means that the central server does not gain access to any individual’s data but only to the collective knowledge learned across the distributed system. The integrity of this process is maintained through secure transmission protocols and cryptography methods, ensuring that updates are not altered. The central server then aggregates the updates from all institutions, creating a global model that benefits from the knowledge learned across the distributed network. The third stage, Model Improvement and Distribution, involves the redistribution of the updated model to each participating institution. Each institution can then use the improved model to make predictions or analyze local data. This process is iterative, with the model continuously improving as new data are collected [14] (Figure 1).

In addition to the basic FL methodology, advanced techniques such as Ontology-Based Data Access (OBDA) and Ontology-Based Data Federation (OBDF) can be used to integrate heterogeneous genomic data sources in a secure and efficient manner. Ontologies are structured frameworks that formally define the concepts, relationships, and properties within a specific domain of knowledge [23]. They are a shared vocabulary, providing a standardized way to represent and communicate complex information across systems and contributors. Ontologies consist of classes (concepts or categories), properties (attributes or relationships), and individuals (instances of classes), which are organized hierarchically and semantically. Ontologies are typically encoded in languages such as OWL (Web Ontology Language), which allows for the use of Description Logic (DL) to support complex queries and knowledge discovery characterizing domains like biomedicine, where data is often intricate and interconnected [24]. For instance, an ontology might define the relationships between genes, phenotypes, diseases, and treatments in genomics, enabling the seamless integration of diverse datasets. Ontologies act as the foundation for systems that need strong data integration, reasoning, and the ability to perform semantic searches. In OBDA, data are semantically aligned through mapping languages like R2RML, enabling SPARQL queries to be automatically translated into SQL [25,26]. This approach bypasses manual data preprocessing and facilitates semantic querying across diverse data sources. For example, a researcher can query the relationship between a genetic variant and its associated phenotypes across multiple databases without centralizing or manually harmonizing the data.

OBDF extends OBDA by introducing a federated architecture integrating multiple heterogeneous data sources under a unified semantic layer [26]. This architecture incorporates a data federation layer that dynamically virtualizes disparate sources, such as relational databases, NoSQL systems, and flat files, without requiring physical data movement. This ensures that sensitive data remain localized, addressing privacy concerns while enabling real-time querying. The federation layer acts as a virtual schema, connecting distributed data sources and facilitating seamless access.

The application of OBDA and OBDF in genomics is valuable for integrating datasets generated from different sequencing platforms. By providing a unified semantic representation, these frameworks enable researchers to identify platform variations, link genetic variants to clinical outcomes, and execute complex queries combining genomic, phenotypic, and clinical data. These capabilities allow insights that are often unattainable using traditional data integration methods.

In contrast to ontology-driven methods, other approaches like data harmonization frameworks and direct query federation systems offer alternative solutions. Data harmonization frameworks focus on standardizing datasets at the schema level, using predefined templates or metadata standards to ensure compatibility [27]. While these methods are less computationally demanding than ontologies, they often lack the semantic richness needed to capture complex biological relationships. On the other hand, direct query federation systems allow distributed queries without requiring semantic alignment. While they are faster and simpler to implement, they may result in less consistent or interpretable results than ontology-based approaches. In genomic FL, the choice between OBDA/OBDF and alternative methods depends on the specific research goals, data complexity, and computational resources available. Ontologies standardize the representation of genomic data, reducing the variability introduced by differences in data sources or sequencing technologies. At the same time, semantic alignment facilitates the retrieval of relevant data for model training, minimizing computational overhead. Furthermore, by virtualizing data access, OBDF ensures that sensitive genomic information remains within its original repository, complying with privacy regulations such as GDPR.

Together, these technologies provide a scalable and secure solution for analyzing genomic data within FL frameworks, paving the way for collaborative research that respects both scientific and ethical standards.

## 4. Key Challenges and Technical Considerations

Although FL holds excellent potential for secure and collaborative genomic data analysis, there are significant challenges in implementing federated networks. Genomic data are heterogeneous and need continuous integration across various platforms and systems. It means addressing differences in data formats, query languages, and computational frameworks. Moreover, another technical issue is ensuring that federated networks can function smoothly through jurisdictions, each with distinct privacy regulations [14].

A critical aspect of the success of FL in genomics is the establishment of standardized Application Programming Interfaces (APIs) and secure computing environments. Standardized interfaces are necessary to ensure that data sources from diverse institutions can be integrated without technical barriers. Secure computing environments are essential to protect sensitive genomic data during model training, mainly when it involves cloud or edge computing infrastructure. Privacy frameworks, such as secure multi-party computation (SMPC) and homomorphic encryption (HE), are also crucial in ensuring that sensitive data is never exposed during analysis. These considerations support data privacy protection while providing meaningful insights into institutional collaboration. SMPC allows multiple parties to perform a computation on their private data inputs without revealing the data to each other. SMPC ensures that no party learns anything more than the result of the computation itself. This makes it highly valuable in scenarios like genomics, where datasets are distributed across multiple institutions. The key advantage is that the data remain decentralized and private while enabling collaborative analysis. HE, on the other hand, enables computations to be performed on encrypted data without decrypting it first. This allows sensitive data to remain encrypted during the entire computation process, thus ensuring privacy even while the data are being processed. HE supports operations like addition and multiplication on ciphertexts, which are then decrypted to reveal the result [28,29]. This technique is particularly useful in federated learning, where data cannot be transferred or decrypted on central servers, ensuring compliance with privacy regulations like GDPR.

FL is not immune to risks such as the re-identification of individuals or cyber-attacks, although it enhances data protection. Re-identification occurs when anonymized data are linked back to an individual, a significant problem when working with sensitive genomic data. Cyberattacks targeting federated networks may potentially reveal weaknesses in distributed systems, compromising data integrity and privacy. To mitigate these risks, encryption methods such as end-to-end encryption and robust authentication protocols, including federated identity management, become increasingly important [30]. These strategies ensure that even if data are intercepted or corrupted, they remain protected from unauthorized access.

## 5. The General Data Protection Regulation Overview

The GDPR, introduced as Regulation (EU) 2016/679, represents a pivotal step in protecting personal data and ensuring privacy rights across the European Union. Effective from 25 May 2018, it replaced Directive 95/46/EC, aiming to harmonize data protection laws across member states [31]. Its core mission is to empower individuals by giving them greater control over their personal data while fostering trust and accountability in the digital economy. At the center of the GDPR lies the principle that personal data must be processed lawfully, fairly, and transparently. Organizations must ensure that data collection and processing are aligned with specific, legitimate purposes and that the amount of data collected is strictly necessary. Transparency is key, as individuals must be informed about how their data are being used, stored, and shared. Furthermore, the GDPR emphasizes the need for accuracy, requiring data controllers to ensure that personal data are kept up to date and to rectify inaccuracies promptly. The regulation also introduces a series of rights for individuals, providing them with more autonomy over their personal information. These include the right to access personal data, the right to rectify or erase data, and the right to data portability, which allows individuals to transfer their data between service providers seamlessly. One of its most prominent features is the “right to be forgotten”, enabling individuals to request the deletion of their data under specific circumstances. In addition to empowering individuals, the GDPR imposes stringent obligations on organizations, which are required to implement robust data protection measures. This includes conducting regular data protection impact assessments, reporting data breaches within 72 h, and appointing Data Protection Officers (DPOs) where necessary. The regulation also applies extraterritorially, meaning it affects any organization processing the data of EU residents, regardless of where the organization is located.

One of the most significant aspects of the GDPR is its strict enforcement mechanism. Non-compliance can result in hefty penalties, reaching up to 4% of an organization’s global annual revenue or EUR 20 million, whichever is higher. These sanctions underscore the EU’s commitment to ensuring that data protection is taken seriously. The GDPR also addresses the processing of sensitive data, such as genetic, health, and biometric data, which are afforded special protection due to their potential impact on fundamental rights and freedoms. This is particularly relevant in sectors like healthcare and research, where data processing must balance innovation with privacy concerns.

Ultimately, the GDPR seeks to protect individuals and aims to facilitate the free movement of data across the EU, creating a unified digital market. By fostering transparency, accountability, and trust, it sets a global standard for data protection in the era of rapid technological advancement and digital globalization.

## 6. Balancing Genomic Data Sharing and Privacy Under the GDPR

The General Data Protection Regulation (GDPR) has significant implications for handling biomedical and genomic data, mainly due to the sensitive nature of such data [32]. Genomic data, which include detailed information about an individual’s genetic makeup, are classified as “special category data” under the GDPR due to their potential to reveal intimate aspects of an individual’s health and identity. As such, the regulation places stricter requirements on the processing and storage of genomic data to ensure privacy and safeguard against misuse. In the context of genomic data, the GDPR mandates that data controllers take extra precautions to ensure data security and transparency, especially when handling identifiable personal data. For genomic datasets, this means that measures must be in place to prevent re-identification, either through technical means such as anonymization or by ensuring that access to the data is appropriately controlled. The regulation also addresses the challenges posed by the potential for re-identification, especially in large-scale genomic studies where individuals could theoretically be identified by linking genomic data with other databases.

The challenges posed by GDPR compliance are exemplified by the Human Cell Atlas (HCA) initiative, a major international project that aims to map every cell type in the human body [33]. As the HCA seeks to make genomic data widely accessible for research purposes, it must navigate the complex interplay between open data sharing and the strict requirements of the GDPR [34]. The initiative highlights the necessity of balancing ethical considerations with legal mandates, ensuring that shared data remain both accessible and protected. For example, the HCA employs advanced anonymization techniques and controlled access policies to comply with GDPR, addressing concerns about the potential for re-identification while facilitating global collaboration. Similar challenges are faced by other large-scale genomic initiatives, where the need to share data across borders further complicates adherence to varying local and international privacy regulations. The HCA underscores the importance of integrating robust data protection strategies into genomic research by navigating these complexities. Its approach provides a framework for other projects striving to balance the imperative for scientific advancement with the responsibility to protect individual privacy under GDPR and similar regulations.

The evolving landscape of data protection regulations in genomics underscores the importance of developing robust governance frameworks that ensure compliance while enabling free data flow for scientific advancement. In this context, FL emerges as a powerful ally that aligns closely with GDPR principles by decentralizing data storage and analysis [7,35]. Instead of transferring raw genomic data across borders, FL keeps data localized while sharing only aggregated model updates, significantly reducing the risk of exposing sensitive information or breaching privacy regulations. This decentralized methodology supports key GDPR principles such as data minimization and purpose limitation while enabling effective collaboration across institutions. FL provides an innovative solution for conducting genomic research on a global scale by enabling the secure training of machine learning models on distributed datasets. It mitigates the complexities of cross-border data sharing while adhering to privacy regulations, thereby revolutionizing international collaboration in genomics. As the regulatory landscape evolves, FL’s ability to bridge the gap between compliance and scientific innovation positions it as a critical tool for advancing global health research and policymaking.

While the GDPR has set a global benchmark for data protection, genomic research often requires cross-border collaboration, necessitating compliance with other regional and international data privacy laws. For instance, in the United States, the Health Insurance Portability and Accountability Act (HIPAA) regulates the privacy and security of health data, including genomic information, by mandating de-identification measures and restricting unauthorized access [36]. Similarly, China’s Personal Information Protection Law (PIPL) imposes stringent requirements for data processing, emphasizing data localization and consent, which directly impact international genomic collaborations [37]. In addition, countries like Australia, under the Privacy Act 1988, have specific provisions for the collection and sharing of health data, requiring transparency and safeguards to mitigate the risk of re-identification [38]. These regulatory frameworks, while distinct, share common principles with the GDPR, such as minimizing data exposure, ensuring lawful processing, and protecting individual privacy rights.

Navigating this complex landscape requires genomic research initiatives to adopt harmonized strategies that respect regional laws while enabling global data-sharing efforts. Federated Learning (FL) offers a unique advantage in this regard: by keeping sensitive genomic data local and sharing only aggregated model updates, FL aligns not only with the GDPR but also with other global data protection standards. This decentralized approach reduces the legal and ethical challenges associated with transferring raw data across jurisdictions, positioning FL as a scalable solution for secure, compliant, and collaborative genomic research worldwide.

## 7. Federated Learning Success Stories

FL has demonstrated remarkable success in advancing genomic research through secure and collaborative data sharing. Numerous international and national initiatives have adopted FL to address the challenges of data privacy, fragmentation, and cross-border collaboration. These efforts underscore the transformative potential of FL in genomics, with applications ranging from basic research to precision medicine. International efforts such as the Global Alliance for Genomics and Health (GA4GH) exemplify the transformative role of federated learning. It was founded in 2013 and develops standards for responsibly collecting, storing, analyzing, and sharing genomic data, facilitating secure data sharing across borders and promoting global collaboration. Other global initiatives are Global Alliance for Genomic Data (GAGD), Beyond 1 Million Genomes and CINECA, which use FL to enable cross-border collaborations in genomics, providing a scalable solution to data-sharing challenges without compromising privacy [39]. A notable example of a national-level initiative is the Canadian Distributed Infrastructure for Genomics (CanDIG), which provides a robust framework for integrating genomic and health data from different provinces across Canada, complying with the country’s strict privacy regulations [40]. In the United States, the Big Data to Knowledge (BD2K) initiative highlights the integration of FL into large-scale genomic data analysis. BD2K fosters international collaboration by developing tools and frameworks for distributed genomic data analysis [41]. Like the CanDIG and BD2K initiatives, the potential use of FL in Europe is shown through well-known platforms such as the European Genome Archive [EGA] and the UK Biobank. The EGA is a human molecular and phenotypic data repository, ensuring the secure storage of genetic and clinical data. By March 2022, EGA had accumulated data from over 4500 research studies worldwide, allowing federated data analysis across Europe [42]. The UK Biobank is one of the world’s largest biomedical resources and uses FL techniques to allow researchers to analyze genetic data from over 500,000 participants around the globe, without centralizing this sensitive information [43]. Another key European initiative is COLLAGENE, a French platform designed to facilitate privacy-preserving collaborative genomic research [44].

Collectively, these initiatives underscore the diverse applications of federated learning in genomic research, addressing complex challenges while upholding data privacy and security on a global scale.

## 8. Discussion and Conclusions

As genomic data become increasingly complex, integrating artificial intelligence (AI) into FL frameworks can enhance predictive analytics and accelerate discoveries in genomics. By combining AI algorithms with FL, researchers can develop more robust and accurate models for genomic data analysis, particularly for tasks such as variant interpretation and precision medicine. This integration could significantly improve the ability to predict patient outcomes or treatment responses based on genomic data [45]. The application of FL holds considerable potential beyond genomics, extending into critical areas such as personalized medicine and clinical trials. Integrating advanced AI techniques and sophisticated data analytics will be pivotal in scaling and optimizing these frameworks. Achieving these goals will require sustained collaboration among diverse stakeholders, the development of standardized APIs, strict adherence to ethical and regulatory standards, and innovative and multidisciplinary education. In fact, as the integration of AI into genomics and precision medicine accelerates, the need for comprehensive education and training in this field has never been more critical. Universities must take the lead in revising and updating their curricula to incorporate the latest AI and machine learning advancements, mainly as they apply to genomic research and medical applications.

Traditional medicine, biology, and laboratory science programs should evolve to include modules on AI-driven data analysis, federated learning, and the ethical and legal considerations of working with sensitive genomic data [46]. Beyond academia, continuous professional development is essential for healthcare professionals, including physicians, biologists, and laboratory technicians. These professionals must be equipped to understand and apply AI tools effectively, ensuring they can harness the full potential of this transformative technology. Just as the discovery of antibiotics revolutionized medicine in the 20th century, AI has the potential to redefine diagnostics, therapeutics, and personalized medicine in the 21st century [47,48,49,50]. However, realizing this potential requires a workforce that is aware of AI’s capabilities and adept at implementing them responsibly and effectively.

Training programs should focus on building practical skills, such as using AI tools for genomic data interpretation, understanding federated learning frameworks, and collaborating within decentralized data networks. Additionally, interdisciplinary approaches that bridge computer science, biology, and healthcare should be encouraged to foster a deeper understanding of how AI and genomics can be combined to address complex medical challenges. By investing in education at all levels, from undergraduate courses to specialized training for active professionals, we can ensure that the healthcare system is prepared to embrace and optimize this revolutionary paradigm shift in medicine [51].

## 9. Future Directions

The future of Federated Learning (FL) in genomics looks promising, with its application potentially extending beyond genomics to other domains such as clinical trials and personalized medicine [52]. For instance, FL could be used to analyze patient data in clinical trials conducted across multiple hospitals, enabling collaboration without transferring sensitive patient data between institutions. This approach would enhance privacy, foster robust large-scale studies, and pave the way for breakthroughs in precision medicine. In addition to these applications, FL holds significant potential for strengthening national and clinical networks. For example, we recently published the result of a multicentric genetic study performed on behalf of the Italian Neuroscience and Neurorehabilitation Network (RIN). RIN is the largest federation of Scientific Institutes for Research, Hospitalization, and Healthcare (IRCCS) in Italy focused on neuroscience. Established in 2017 by the Italian Ministry of Health, its mission is to promote collaboration among IRCCS centers, facilitate the exchange of clinical and scientific data, and coordinate the development of protocols and algorithms for translational research. The network supports scientific and technological advancements in the prevention, diagnosis, treatment, and rehabilitation of neurodegenerative, neurological, neuropsychiatric, and related disorders. Given the complexity and diversity of neurological conditions, research efforts are divided into specific thematic areas. To achieve this, RIN has created Virtual Institutes of Pathology (VIP), each dedicated to specific diseases or groups of conditions, such as dementia, movement disorders, immunological diseases, motor neuron diseases, epilepsy, cerebrovascular disorders, neuro-oncology, and rare neurological diseases [53].

Every VIP could leverage FL to enable secure collaboration among institutions studying neurological diseases and rehabilitation.

By allowing the training of machine learning models on distributed datasets without centralizing sensitive information, FL would help overcome the challenges posed by the small sample sizes and fragmented data typical in these fields. This approach could accelerate the identification of key biomarkers and support the development of new therapies for neurological conditions. Similarly, the Facioscapulohumeral Muscular Dystrophy (FSHD) Italian Clinical Group presents another promising use case. The group focuses on studying this rare muscular dystrophy through the integration of genomic, phenotypic, clinical and imaging data from multiple centers [54]. FL could facilitate the development of predictive models for genotype–phenotype correlation, disease progression and therapy response while preserving patient confidentiality. By addressing these specific challenges, FL can enhance collaboration within clinical and research consortia, fostering advancements in both rare disease research and broader genomic applications.

These examples highlight the transformative potential of FL in addressing privacy concerns and enabling collaboration in genomics and beyond. As FL continues to evolve, its integration into national and international consortia will provide a roadmap for expanding its use across diverse fields, revolutionizing how sensitive biomedical data are utilized to advance scientific discovery and improve patient outcomes.

## Figures and Tables

**Table 1:** Comparison of data management approaches in genomics: centralized, decentralized, and federated learning systems.

	Centralized	Decentralized	Federated Learning
Definition	Data are collected and stored in a single central location.	Data remain at their origin and are analyzed locally.	Data remain at their origin, and a model is trained locally.
Data Transfer	Complete transfer of data to a central server is required.	No complete transfer; only results from local analyses are exchanged.	No data transfer; only model parameters are shared.
Security and Privacy	Higher risk of privacy breaches due to centralized storage of the data.	Data remain local, so the security depends on the local infrastructure.	Depends on the infrastructure and privacy techniques, like encryption, during model training.
Scalability	Limited by the capacity of the central server.	Moderate scalability; depends on the network setup.	Highly scalable as computation is distributed.
Latency	Potential bottlenecks due to central processing.	Reduced latency as processing is local.	Minimal latency as only updates are transmitted.
Advantages	Easier to manage in one location.	More control is required for data owners, but it requires secure infrastructure.	Combines privacy, security, and scalability for efficient collaboration.

## Data Availability

No new data were created or analyzed in this study.
